# Effects of Local Anthropogenic Changes on Potential Malaria Vector *Anopheles hyrcanus* and West Nile Virus Vector *Culex modestus,* Camargue, France

**DOI:** 10.3201/eid1312.070730

**Published:** 2007-12

**Authors:** Nicolas Ponçon, Thomas Balenghien, Céline Toty, Jean Baptiste Ferré, Cyrille Thomas, Alain Dervieux, Grégory L’Ambert, Francis Schaffner, Olivier Bardin, Didier Fontenille

**Affiliations:** * Institut de Recherche pour le Développement, Montpellier, France; † École Nationale Vétérinaire de Lyon, Marcy l’Étoile, France; ‡ Centre de Coopération Internationale en Recherche Agronomique pour le Développement, Montpellier, France; § Entente Interdépartementale pour la Démoustication Méditerranée, Montpellier, France; ¶ Centre Français du Riz, Arles, France; # Centre National de la Recherche Scientifique, Arles, France; ** University of Zürich, Zürich, Switzerland

**Keywords:** malaria, West Nile virus, Anopheles, Culex, France, environment, perspective

## Abstract

Using historical data, we highlight the consequences of anthropogenic ecosystem modifications on the abundance of mosquitoes implicated as the current most important potential malaria vector, *Anopheles hyrcanus,* and the most important West Nile virus (WNV) vector, *Culex modestus*, in the Camargue region, France. From World War II to 1971, populations of these species increased as rice cultivation expanded in the region in a political context that supported agriculture. They then fell, likely because of decreased cultivation and increased pesticide use to control a rice pest. The species increased again after 2000 with the advent of more targeted pest-management strategies, mainly the results of European regulations decisions. An intertwined influence of political context, environmental constraints, technical improvements, and social factors led to changes in mosquito abundance that had potential consequences on malaria and WNV transmission. These findings suggest that anthropogenic changes should not be underestimated in vectorborne disease recrudescence.

## Abstract 2

Sixty years of environmental modifications have led to strong and rapid effects on the abundance of vector populations.

During the past 25 years, there has been a dramatic emergence and resurgence of epidemic vectorborne diseases affecting both humans and domestic animals (*1*). In most cases, sociodemographic changes, drug resistance, and anthropogenic environmental modifications appear to be the main factors responsible (*1*–*4*). The Camargue, the Rhone River Delta region in southeastern France, is an area relevant to the study of the influence of environmental changes on vector populations because 1) it has witnessed important anthropogenic ecosystem modifications in the past 60 years, 2) it contains a great abundance and diversity of mosquito-breeding sites and thus hosts large mosquito populations, and 3) it is a former zone of endemic malaria and a region of current and regular transmission of West Nile fever.

Until the beginning of the 20th century, malaria, mainly transmitted by *Anopheles (Anopheles) atroparvus* Van Thiel, was endemic in the Camargue and constituted a major health issue there (*5*,*6*). The last *Plasmodium vivax* malaria epidemic occurred in 1943, with ≈400 cases (*7*). Malaria disappeared from this area after World War II because of improved housing and living conditions and the extensive use of quinine. Among 8 anopheline species recorded in the Camargue, *An. atroparvus* was recently found to be rare and *An. (Anopheles) hyrcanus* (Pallas) very abundant with a high human-biting rate (*8*), findings that suggest that *An. hyrcanus* is currently the only *Culicidae* sp. likely to play a role in malaria transmission in the Camargue (*8*). Moreover, autochthonous transmission was recently suspected on the French Mediterranean Coast in 2006 (*9*), which also supports the idea that southern France remains suitable for malaria transmission.

The first description of West Nile virus (WNV) in France was in the 1960s, with human and equine outbreaks in the Camargue (*10*). After these episodes, the disease seemed to disappear from this region. However, WNV transmission apparently continued thereafter and was confirmed by serologic studies in the 1970s and 1980s (*11*,*12*). Since 2000, WNV-related disease has reappeared in southern France, causing equine outbreaks in 2000 (76 confirmed cases) and 2004 (32 confirmed cases) in the Camargue (hosting 7,000 equids) and sporadic human and/or equine cases in 2003 and 2006 elsewhere along the Mediterranean Coast (*13*–*16*). Among 7 *Culex* species recorded in the Camargue (*17*), *Culex (Barraudius) modestus* Ficalbi is considered the main WNV vector, based on abundance, feeding behavior, previous WNV isolations, and recent experimental transmission (*18*–*20*).

The aim of this article is to describe the history of the region and to examine the impact of the various anthropogenic environmental changes that have occurred in the Camargue over the past 60 years on 2 mosquito species, *An. hyrcanus* and *Cx. modestus.* Because rice fields are the quasi-exclusive breeding sites for *An. hyrcanus* and the most prolific sites for *Cx. modestus* (*5*), we focus on changes in rice cultivation, i.e., cultivated surfaces and agronomic practices, including insecticide spraying, related to socioeconomic and agronomic factors.

## Context

### The Study Area

**Figure 1:**
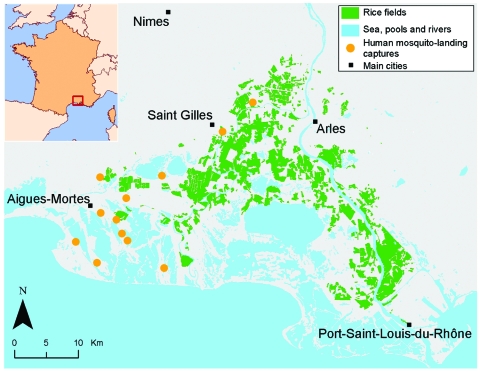
Map of the Camargue, France, indicating areas of rice cultivation as well as mosquito sampling sites, 2001.

The Camargue is the main wetland area in the southeast of France and covers the Rhone River Delta (Figure 1). This area has a Mediterranean climate characterized by warm, dry summers and mild, wet winters. Total annual rainfall is typically 500–700 mm and occurs mainly in autumn; the annual mean temperature is 14°C.

Landscapes in the Camargue are strongly affected by the duration of submersion and the salinity of the soils. The landscapes are organized roughly in a south-to-north gradient of salinity, with agricultural land and reed marshes in the north and natural salty ponds and salt marshes in the south. Most agricultural land belongs to a few large farms, which are able to rapidly change their production system (i.e., crop type), depending on the economic context (*21*,*22*). Rice is currently the main cultivated crop in the Camargue, which is almost the only French region that produces rice. Paddies are filled in April and May with ≈7 cm of water. From the end of June until the end of August, a depth of ≈20 cm of water is maintained in the paddies, and the rice plants cover their surfaces. The water is then drained and the rice harvested. Data on rice cultivation used in this article were provided by the French National Rice Center.

### The Mosquito Species

*A. hyrcanus* is a Palearctic mosquito species belonging to the Hyrcanus group. It is distributed from Spain to People’s Republic of China, covering the southern half of Europe, the Mediterranean area, and central Asia. Large populations are found in irrigated rice-growing areas in Turkey, Greece, and France (*23*,*24*), and this species was involved in malaria transmission in the north of Afghanistan (*25*).

*Cx. modestus* is also a Palearctic species, widely distributed from Europe to India, especially in delta areas, where its larvae can be found in semipermanent reed marshes, irrigation canals, and rice fields (*5*). The involvement of *Cx. modestus* in WNV transmission was established in the Camargue (*20*,*26*), the delta areas of the Caspian and Azov Seas (*27*,*28*), and the Volga region of Russia (*29*) and was suspected in the Danube Delta (*30*).

For our study, changes in *An. hyrcanus* and *Cx. modestus* abundance were assessed by using 1) literature data, 2) detailed annual activity reports that describe nuisance caused by mosquito pests and published from 1962 to 1996, and 3) data from regular human mosquito-landing collections conducted since 1969 in 12 sites in the western Camargue (for 15 minutes at sunrise, approximately once a week from June to October). These sites were sampled by using the same methods, thus allowing us to describe changes in *An. hyrcanus* and *Cx. modestus* abundance over a 38-year period. Collection sites were distributed in the western Camargue, which allowed a sampling of all ecosystems from the north to the south, reflecting mosquito abundance in the entire region (Figure 1). This human mosquito-landing survey did not focus specifically on *An. hyrcanus* and *Cx. modestus.* Thus, some sites were located some distance from the indicated area under cultivation (even if these areas have changed during the past 60 years) and were probably always negative for *An. hyrcanus* and *Cx. modestus*. Unfortunately, results of human mosquito-landing collections were not available per sample site. Thus, to avoid overrepresentation of uninformative and consistently negative sites, quantitative abundance of *An. hyrcanus* and *Cx. modestus* was assessed by the annual mean number of mosquitoes caught by positive collection. Changes in rice cultivation and mosquito populations, shown in the Appendix Figure, were analyzed for 3 periods, described below.

## Changes in Mosquito Populations and Rice Cultivation since the 1920s

### 1920s to 1960s: Proliferation of *An. hyrcanus* and *Cx. modestus*

In the Camargue, rice cultivation was rare before World War II, and both *An. hyrcanus* and *Cx. modestus* were only rarely reported after their first description in the 1920s until World War II (*5*,*31*–*36*). The development of rice cultivation started with the rice shortage caused by World War II and was supported by a guaranteed price and funds from the Marshall Plan in 1947 and by the agricultural equipment cooperative established in 1948. Rice cultivation was then mechanized and hugely increased to cover ≈30,000 ha during the 1960s (Appendix Figure). In the 1950s and the 1960s, *An. hyrcanus* and *Cx. modestus* populations were described as widely distributed and very abundant in the entire Camargue, and these 2 species were included in the group of the 3 most abundant nuisance biters (*5*). *An. hyrcanus* was considered a major pest in the western Camargue in 1969 and 1970, and *Cx. modestus* attacks reached 300 bites per person per hour in reed marshes (*26*). From 1942 to the 1960s, the increase in *An. hyrcanus* and *Cx. modestus* populations seemed to follow changes in paddy surface area (Appendix Figure), itself a product of the political consequences of World War II (e.g., agricultural support, mechanization).

### 1960s to 1999: Near Disappearance of Mosquito Populations

#### Decrease of Area under Rice Cultivation

In 1963, the enforcement of the Common Agricultural Policy of the European Community caused unfavorable conditions for French rice cultivation, which was confronted by the more competitive Italian rice cultivation. This situation depressed both prices and incomes for French producers, who abandoned rice cultivation and developed alternatives such as hard wheat. The area under rice cultivation started to decrease slowly after 1965 (Appendix Figure).

#### Insecticide Implementation

In 1970, the striped rice borer, *Chilo suppressalis* (Walker), a pest insect that damages rice plants, was introduced into France on young rice plants imported from Spain (*37*). From 1972, rice producers implemented insecticide sprayings with fenitrothion, trichlorfon, and chlorphenamidine, which were conducted at the end of July each year, to control this pest (Table). The striped rice borer invasion reduced French rice competitiveness and consequently accelerated the decline in rice cultivation to 4,400 ha by 1981.

**Table:** Sprayed rice surfaces (hectares) to control striped rice borer in the Camargue

Years of insecticide spraying	Insecticides effective against mosquitoes			Lepidopteron-specific insecticides	
	Fenitrothion, trichlorfon, and chlorphenamidine	Alphamethrin		*Bacillus thuringiensis kurstaki*	Tebufenozide
1972	10,000	0		0	0
1973	6,500	0		0	0
1974	9,000	0		0	0
1975–1989	Very limited	0		0	0
1990–1996	0	Intensive, ≈2/3 of rice surfaces		0	0
1996–1999	0			Permitted but not often used	0
2000	0	11,500		500	0
2001	0	11,500		500	200
2002	0	10,000		2,000	300
2003	0	10,000		2,000	400
2004	0	4,500		1,000	500
2005	0	0		800	2,300
2006	0	0		Very limited	≈3,000

Human mosquito-landing collections showed a drastic drop in *An. hyrcanus* and a progressive decrease in *Cx. modestus* populations in 1972 and 1973, after the insecticide sprayings were initiated (Appendix Figure). At the end of July, insecticide, also efficient against mosquito larvae, was sprayed by fixed-wing airplane that used low-volume applications (15 L/hectare); the insecticide reached the water even when rice plants covered the paddy surfaces. At this time of year, *An. hyrcanus* and *Cx. modestus* larvae usually massively colonize rice fields, which in summer are nearly the only available breeding sites for these species (N. Ponçon, unpub. data) (*26*). These sprayings likely reduced *An. hyrcanus* populations considerably, with the removal of water from paddies at the end of August limiting posttreatment population recovery. In September, flooding of reed marshes, which are natural breeding sites for *Cx. modestus*, allows only a limited maintenance of populations and probably explains the slower decrease of this species than of *An. hyrcanus*. Indeed, reed marshes cannot maintain important populations, as illustrated by the rareness of *Cx. modestus* before World War II.

#### Increase of Area under Rice Cultivation and of Insecticide Sprayings

In 1981 a French support plan was implemented that led to an increase in rice cultivation, which covered >20,000 ha by the early 1990s. In 1994 the General Agreement on Tariffs and Trade limited subsidies, and French rice cultivation, still fairly uncompetitive, experienced difficulties once again. These problems were accentuated by a new demand for perfumed rice varieties that are not produced in the Camargue. Some producers thus replaced rice with hard wheat, which explains the decrease in rice cultivation areas since 1994 (Appendix Figure).

In 1988 a new rice variety, *Ariete*, was introduced into the Camargue and, from 1991 to 2000, it quickly became the most cultivated rice. This variety of rice is very susceptible to the striped rice borer. Consequently, producers sprayed large areas to avoid losses and to ensure high productivity. Sprayings were conducted by using the same methods as before except that alphamethrin, also efficient against mosquito larvae, replaced the former insecticides.

The intensive insecticide sprayings against the striped rice borer likely account for the low populations of *An. hyrcanus* and *Cx. modestus* over this period, despite the increase in the area of rice cultivation. Human mosquito-landing data showed a slight population peak in both species in 1994, when rice cultivation covered a maximum of 24,500 ha (Appendix Figure).

### From 2000: Increase in Mosquito Populations

More recently still, rice producers have developed other cultivations in rotation with rice and have added new activities, such as hunting marshes and tourism, with the aim of diminishing their dependence on rice economics (*22*). Since 2000, the area under rice cultivation has remained stable at ≈18,000–20,000 ha.

Since 2000 the *Ariete* variety of rice has been progressively replaced by varieties less susceptible to the striped rice borer. Consequently, spraying was conducted over smaller percentages of the rice-cultivated areas: 61% in 2000 to 51% in 2003. Nevertheless, spraying was maintained to ensure the high productivity on which subsidies were based at that time. Since 2004, the terms of rice subsidies have changed yet again, leading to the disinterest in high productivity and to the high decrease in sprayed surfaces in 2004. Finally, controls on insecticide use were strengthened in 2005 (departmental order, Mar 5, 2004) to limit the impact on wild fauna; the use of alphamethrin by airplane was abandoned, and the emphasis now is on lepidopteron-specific insecticides (tebufenozide, *Bacillus thuringiensis kurstaki 3a*/*3b*). In parallel with the progressive abandon of insecticide, *An. hyrcanus* and *Cx. modestus* populations have increased continuously from 2000 to the present.

## Untangling the Components of Anthropogenic Change

Before the 1970s, and in the absence of insecticide spraying, *An. hyrcanus* and *Cx. modestus* abundance followed the increase in the area under rice cultivation. After 1970, insecticide spraying, which was aimed at controlling the striped rice borer, likely influenced the size of the mosquito populations. From 1972 to 1974 and from 2000 to 2006 (surfaces sprayed were precisely known only during these years), the abundance of both mosquito species (human mosquito-landing data) was negatively correlated with the percentage of the rice areas sprayed with mosquito-efficient insecticides (Pearson coefficient r = –0.84, p<0.001 for *An. hyrcanus* and r = –0.64, p<0.05 for *Cx. modestus*).

**Figure 2:**
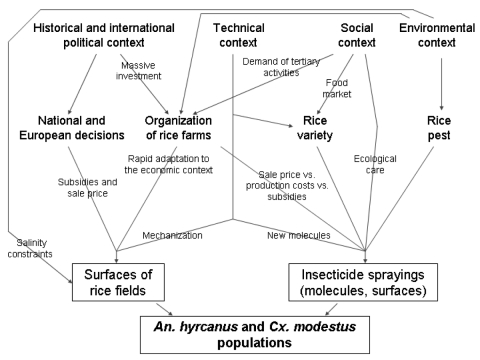
Impact of history, politics, technology, society, and environment on malaria and West Nile fever in the Camargue, France.

This story highlights the intertwined importance of historical, political, environmental, technical, and social factors in explaining agricultural changes in the Camargue that could have directly contributed to variation in the abundance of both *An. hyrcanus* and *Cx. modestus* populations, with possible consequences for vectorborne diseases (Figure 2). *An. hyrcanus* is currently considered the main potential malaria vector in the Camargue, whereas the past periods of high *Cx. modestus* abundance, i.e., the 1960s and the 2000s, were associated with WNV outbreaks in the Camargue.

The amount of rice cultivation in this area was determined by national or European decisions, which were influenced by the global historical and political context. Favorable economic conditions for rice cultivation (Marshall Plan, guaranteed prices, and subsidies) were the results of the World War II and the developing Cold War; later economic globalization forced a decrease in this support. The close relationship between political decisions and variations in rice surface area in the Camargue is due to the organization of farming into large units, itself a product of past massive funding investments and environmental constraints; this system allowed a rapid response to the changing economic climate. Currently, rice producers in the Camargue are adding tertiary activities to their historical role as food providers, in response to new social demands concerning leisure such as hunting and nature tourism. The extent and amount of spray applied depends on the presence of rice pests, permissiveness of the rice variety; and the insecticide cost in regard to production costs, sale price, and subsidies (indexed or not on productivity). The choice of rice variety is determined by its adequacy within the food market and its agronomic performance in the production area.

What does the future hold for these mosquito populations in the Camargue? On the one hand, the Common Agricultural Policy will face another round of debates about subsidies in 2013. If subsidies are reduced, rice cultivation is expected to decrease; *An. hyrcanus* and *Cx. modestus* will therefore also likely decrease in abundance and may even become as rare as they were before World War II. Conversely, because rice cultivators are important actors in maintaining the ecosystems of the Camargue, the French government may decide to continue to support rice cultivation there. Without any other disturbance of this ecosystem, *An. hyrcanus* and *Cx. modestus* populations might then continue to increase.

Climate change is considered by some authors as being responsible for the vectorborne disease recrudescence (*38*,*39*). However, as our data indicate, environmental modifications and changes in the economic, social, and cultural environments can have strong and rapid effects on mosquito populations.

## Supplementary Material

Appendix Figure Changes in vector abundance and rice cultivation related to economic and agronomic contexts in the Camargue, France. Ha, hectares. Rice surface, insecticide-sprayed surface, and human mosquito-landing collection data were smoothed with a centered moving average (running mean) of 3 years to filter short-term variations. *Insecticide sprayed surface, only insecticides having an effect on mosquito larvae (i.e., fenithrotion, trichlorfon, chlorphenamidine, and alphamethrin) were included; surfaces sprayed with lepidopteron-specific insecticide (i.e., tebufenozide and *Bacillus thuringiensis kurstaki*) were not included. Sprayed surfaces were precisely known from 1972 to 1974 and from 2000 to 2006 and were estimated from 1990 to 1999. ‡*Anopheles hyrcanus* is the main potential malaria vector, and *Culex modestus* is the main West Nile virus vector in the Camargue. §No data on sprayings, years 1975–1989, for which no quantitative data on sprayed surfaces were available.
