# Supplementary figures and images for: Effects of Local Anthropogenic Changes on Potential Malaria Vector Anopheles hyrcanus and West Nile Virus Vector Culex modestus, Camargue, France
Source: Emerg Infect Dis. 2007 Dec;13(12):1810–5. doi: 10.3201/eid1312.070730 (PMC2876767; doi:10.3201/eid1312.070730)

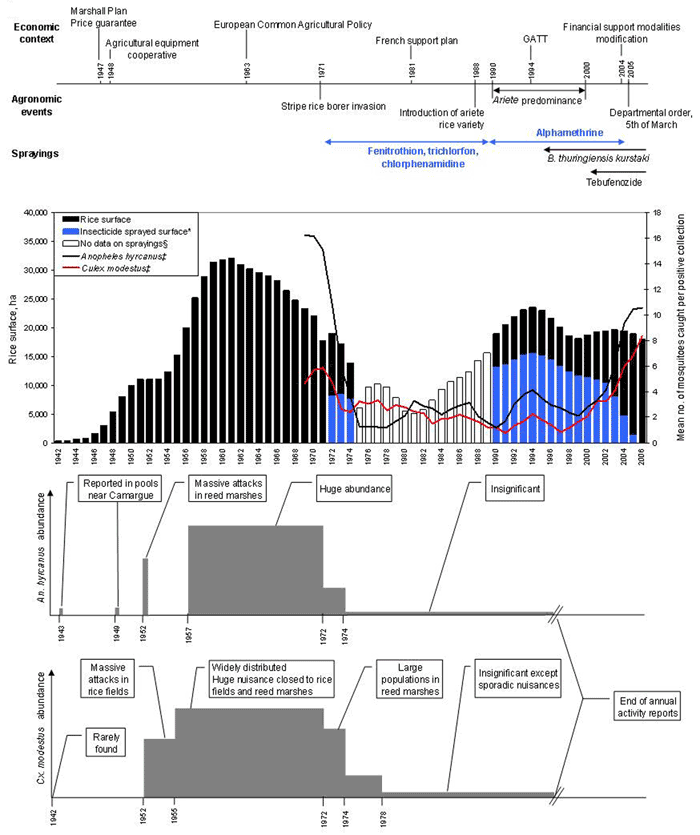

Supplement: Appendix Figure — Changes in vector abundance and rice cultivation related to economic and agronomic contexts in the Camargue, France. Ha, hectares. Rice surface, insecticide-sprayed surface, and human mosquito-landing collection data were smoothed with a centered moving average (running mean) of 3 years to filter short-term variations. *Insecticide sprayed surface, only insecticides having an effect on mosquito larvae (i.e., fenithrotion, trichlorfon, chlorphenamidine, and alphamethrin) were included; surfaces sprayed with lepidopteron-specific insecticide (i.e., tebufenozide and Bacillus thuringiensis kurstaki) were not included. Sprayed surfaces were precisely known from 1972 to 1974 and from 2000 to 2006 and were estimated from 1990 to 1999. ‡Anopheles hyrcanus is the main potential malaria vector, and Culex modestus is the main West Nile virus vector in the Camargue. §No data on sprayings, years 1975–1989, for which no quantitative data on sprayed surfaces were available. [file 07-0730_appF-s1.gif]
